# Estimating the COVID-19 prevalence and mortality using a novel data-driven hybrid model based on ensemble empirical mode decomposition

**DOI:** 10.1038/s41598-021-00948-6

**Published:** 2021-11-01

**Authors:** Yongbin Wang, Chunjie Xu, Sanqiao Yao, Lei Wang, Yingzheng Zhao, Jingchao Ren, Yuchun Li

**Affiliations:** 1 Department of Epidemiology and Health Statistics, School of Public Health, Xinxiang Medical University, No. 601 Jinsui Road, Hongqi District, Xinxiang City, 453003 Henan Province People’s Republic of China; 2 Department of Occupational and Environmental Health, School of Public Health, Capital Medical University, Beijing, People’s Republic of China; 3 Center for Musculoskeletal Surgery, Charité–Universitätsmedizin Berlin, Freie Universität Berlin, Humboldt–Universität zu Berlin and Berlin Institute of Health, Berlin, Germany

**Keywords:** Infectious diseases, Disease prevention

## Abstract

In this study, we proposed a new data-driven hybrid technique by integrating an ensemble empirical mode decomposition (EEMD), an autoregressive integrated moving average (ARIMA), with a nonlinear autoregressive artificial neural network (NARANN), called the EEMD-ARIMA-NARANN model, to perform time series modeling and forecasting based on the COVID-19 prevalence and mortality data from 28 February 2020 to 27 June 2020 in South Africa and Nigeria. By comparing the accuracy level of forecasting measurements with the basic ARIMA and NARANN models, it was shown that this novel data-driven hybrid model did a better job of capturing the dynamic changing trends of the target data than the others used in this work. Our proposed mixture technique can be deemed as a helpful policy-supportive tool to plan and provide medical supplies effectively. The overall confirmed cases and deaths were estimated to reach around 176,570 [95% uncertainty level (UL) 173,607 to 178,476] and 3454 (95% UL 3384 to 3487), respectively, in South Africa, along with 32,136 (95% UL 31,568 to 32,641) and 788 (95% UL 775 to 804) in Nigeria on 12 July 2020 using this data-driven EEMD-ARIMA-NARANN hybrid technique. The contributions of this study include three aspects. First, the proposed hybrid model can better capture the dynamic dependency characteristics compared with the individual models. Second, this new data-driven hybrid model is constructed in a more reasonable way relative to the traditional mixture model. Third, this proposed model may be generalized to estimate the epidemic patterns of COVID-19 in other regions.

## Introduction

In December 2019, a type of new pneumonia of unknown etiology initially occurred in the city of Wuhan, China, and soon afterward, Wuhan became the epicenter of the outbreak of this disease, later named as coronavirus disease 2019 (COVID-19) caused by the severe acute respiratory syndrome coronavirus 2 (SARS-CoV-2)^1,2^. Since then, COVID-19 has been bombarding almost every corner of the world for just two months and has become a universal pandemic^3,4^. COVID-19 is highly contagious and has caused a series of massive negative effects on economic progress, people’s lives and health around the globe, and it has been identified as being the foremost global public health crisis since the twentieth century^5,6^. As of June 27, 2020, the outbreak has resulted in a great tragedy with overall 9,653,048 confirmed cases and 491,128 deaths in more than 200 countries of our planet^2^. The current reported cases and deaths may be underestimated in the seriously affected regions to a great extent as there are limited medical and health resources that satisfy the requirement of the epidemiological surveillance and detection^7^, and it is estimated that the present epidemiological trend may still be rising exponentially in the near future^2^. Such an emergency has raised many significant issues associated with the spreading dynamics, the alleviation, along with the response strategies and measures of this public health emergency of international concern. Unfortunately, because of the new nature of the SARS-CoV-2, there is still an absence of enough knowledge regarding this virus and an absence of clinical treatment determined and vaccines available, leading to greater uncertainty in the decision-making process. In this scenario, an accurate estimate based on mathematical and statistical techniques can provide a basis for the formulation of effective planning to better tackle the societal, economical, cultural, and public health issues related to this pandemic^8,9^. Also, it is extremely crucial for directing the intensity and type of interventions required to mitigate this public health emergency^10^.

Time series analysis is significantly instrumental in understanding the past epidemic patterns of the diseases and in forecasting the upcoming epidemiological trends based on the past and current inherent rules of the target series by adopting different modeling methods^4,7,11^. Over the past decades, different time series modeling techniques with high reliability levels have been employed for various forecasting purposes. More recently, a large and growing body of literature has investigated the usefulness of the statistical methods to forecast the transmission of the COVID-19 outbreak in order to serve as a reference for mitigating the outbreak, and some of which have played an important role in containing the spread of the COVID-19 outbreak. For example, many current prevention and control measures (e.g., keeping social distancing, wearing face masks, isolation, and observation of the cases and close contacts, the establishment of mobile cabin hospitals, lockdown of the area or countries, travel restrictions and border control, and human mobility restrictions) are formed based on the resulting results of model forecasting^4,12–18^. The current common use of the modeling methods includes the autoregressive integrated moving average (ARIMA) model^4,7,19–24^, genetic programming^25^, simple model of growth^26^, support vector regression^27^, unbiased hierarchical bayesian estimator approach^28^, susceptible-exposed-infected-recovery (SEIR) model^28^, linear regression models^29^, and stereographic Brownian diffusion epidemiology model (SBDiEM)^30^. Time series data are often restricted and affected by many potential determinants, leading to showing complicated linear and nonlinear interaction, together with non-stationarity in the data^31^. For this reason, the mentioned methods failed to take full advantage of these components simultaneously as they are under the linear or nonlinear assumption, and therefore the results from them are difficult to be generalized. To improve the forecasting reliability level, an alternative approach should be tailored for use with both tendencies (linear component) and randomness (nonlinear component). Motivated by this idea, researchers have developed hybrid models by integrating linear models with nonlinear models (e.g., ARIMA-generalized regression neural network [GRNN], ARIMA– backpropagation neural network [BPNN], and autoregressive [AR]-time delay neural network [TDNN] hybrid models)^32–34^, which may generate better forecasting by use of each method’s capability. In such traditional ensemble architectures, the ARIMA or AR model is often used to capture the linear dependency structure in a time series, and then the residuals of a linear pattern is assumed to include the nonlinear component that can be captured by the neural network models (ANN_S_)^34,35^. However, such an assumption may lead to an underestimation of the relationship between the linear and nonlinear patterns in a time series because the association between these two patterns may fail to be additive^32^. Moreover, the residuals from the linear models may not comprise valid non-linear component in a time series^32^. Importantly, recent published papers have also demonstrated that the traditional mixture methods do not necessarily provide a performance improvement over the individual methods^32,35,36^. For this reason, the challenge for developing a perfect hybrid prediction model is how to identify the underlying linear and nonlinear patterns in a time series.

Wavelet analysis has attracted much attention as a flexible and useful tool able to diagnose high-frequency traits and to extract worthy information especially when time series is characterized by non-stationarity and non-linearity because this analysis has a powerful potential to discern exceptional events by time-localized frequency analysis^4,37,38^. More recently, researchers have developed a novel wavelet decomposition technique-ensemble empirical mode decomposition (EEMD) based on the empirical mode decomposition (EMD) for filtering and handling time series preliminarily, which is capable of overcoming the mode mixing weaknesses of the EMD^39,40^. Unlike the conventional discrete wavelet transform methods that require and predetermine basis functions, causing different decomposition results, EEMD is a self-adaptive, empirical, direct, and intuitive data processing technique, particularly appropriate for handling the non-stationary and non-linear data patterns^41,42^. And many hybrid models that adopt a combination of the EEMD and some algorithms have produced satisfactory results in the time series forecasting field. For instance, Zhou et al. built a mixture model by combining the EEMD and a general regression neural network to predict the PM_2.5_ concentrations^43^. Wang et al. constructed an EEMD decomposition-based ARIMA to improve the prediction reliability level of the annual runoff time series^41^. Wang et al. applied the backpropagation network model based on EEMD decomposition to hydrological time series in order to improve the medium and long-term forecasting accuracy level^41^. However, the above-referenced models are only a simple ensemble architecture comprising either a basic linear or nonlinear model based on the EEMD technique, which is unable to consider both linear and nonlinear components in a time series simultaneously despite a performance improvement over the basic models by use of these ensemble architectures. Motivated by the “decomposition and ensemble” idea based on the EEMD method, a promising alternative is to develop an ensemble architecture by integrating the linear trait with the nonlinear trait decomposed by the EEMD method using an adequate linear model and nonlinear model^44^. By doing so, this new ensemble architecture is capable of capturing both components in a time series simultaneously.

In time series forecasting, the ARIMA model is the most used method to handle linear information, whereas ANN_S_ methods are adept at solving nonlinear problems, and the nonlinear autoregressive artificial neural network (NARANN) model has been demonstrated to have excellent mimic and prediction performances among ANNs models because this model has embedded memory function with the help of the tapped delay lines^45^. Therefore, the present study developed a novel mixture prediction model by considering the respective superiority of the EEMD, ARIMA, and NARANN in addressing time series forecasting issues to estimate the epidemiological trends of the COVID-19 prevalence and mortality in South Africa and Nigeria, the hardest-hit two countries with the outbreak in Africa^2,46^. Specifically, first, applying the EEMD technique to decompose the daily prevalence and mortality series into several Intrinsic Mode Functions (IMFs) subseries together with a residue subseries representing the trend of the data. Second, the IMFs terms were modeled using appropriate NARANN methods, whereas the residue term was modeled with a suitable ARIMA model. Finally, the prediction results from our proposed hybrid model were obtained by a conjunction of those from the basic NARANN and ARIMA models^44^. Since the lack of adequate health infrastructure and services in many regions of Africa, such estimates can elucidate the spreading dynamics of the outbreak, which will be a useful aid for government institutions and policymakers to plan the number of additional materials and resources in order to keep the outbreak under control well. Additionally, such estimates may also assist local people to lessen their present socioeconomic and psychosocial pressures and distresses related to the COVID-19 pandemic.

## Material and methods

### Data source

This research focused on the daily time series analysis of the COVID-19 prevalence and mortality, the overall diagnosed COVID-19 cases and death tolls between 28 February 2020 and 27 June 2020 were taken from the COVID-19 Data Repository by the Center for Systems Science and Engineering (CSSE) at Johns Hopkins University (https://github.com/CSSEGISandData/COVID-19) and the COVID-2019 situation reports by the WHO (https://www.who.int/emergencies/diseases). Often, at least 50 observations and preferably 100 observations or more are required in order to construct an adequate and effective model^47^. Thus, the datasets used in this study were divided into two parts. The subset from 28 February 2020 through 15 June 2020 was treated as the training horizon (109 observations), the other was deemed as the prediction horizon (12 observations).

The study protocol was approved by the research institutional review board of the Xinxiang Medical University (No: XYLL-2019072). All relevant guidelines were followed for the study. Ethical approval is not warranted for this research as these data without personal information are publicly available around the globe and the same is approved by the CSSE and WHO.

### ARIMA model

The ARIMA model has been the most frequently used forecasting tool in the domain of health care in the past because of its simple structure, flexible applicability, and potential to interpret a given time series^7^. Supposing that there exists a certain linear pattern between the past observations and the future observations, the ARIMA model can then make use of this pattern to predict the epidemic trends in the near future^4,48^. A representative ARIMA (p, d, q) model is composed of three components, where, p, d, and q represent the orders of the autoregressive method (AR), the non-seasonal differenced degrees, and the moving average method (MA), respectively. The ARIMA model is often established through four steps. Initially, an augmented Dickey–Fuller (ADF) test was applied to the original data to investigate its stationarity, if indicating a non-stationary series, a differenced transformation would help to achieve stationarity^48,49^. Secondly, the crude values of the key parameters (p, d, and q) were determined by plotting the autocorrelation function (ACF) and partial ACF (PACF) graphs based on the differenced series. Among all the candidate models, the one that produced such goodness of fit measures as a larger value of the log-likelihood, as well as a lower value of the Akaike information criteria (AIC), consistent AIC (CAIC), and Bayesian information criterion (BIC), was considered the preferred^50^. Thirdly, using statistical-based diagnostic indices, including Ljung-Box Q test, ACF plot, PACF plot, and t-test, to check the adequacy of the identified model, once the residuals behaved like a white-noise series under the Ljung-Box Q test and the determined parameters were statistically significant under the t-test, meaning that this model is suitable^51^. Ultimately, the preferred ARIMA method can be employed to conduct out-of-sample forecasts.

### NARANN model

ANNs can well enable arbitrarily complex non-stationary series to obtain any desired accuracy thanks to its flexible nonlinear mapping ability^52^. The NARANN method with the time-varying state of interconnected neurons is an important dynamic recurrent ANNs model. For this reason, this method has the inherent attributes of ANNs (e.g., powerful nonlinear mapping capacity, self-learning and adaption ability, along with generalization and fault-tolerant ability)^33,53^. Further, the NARANN model also has a long or short-term memory function by retaining the prior inputs, outputs and network structures with the help of the tapped delay line, resulting in a dynamic modeling potential to the time-dependent series^33^. An NARANN method can be in the form below

1 $$X_{t} = f(x(t - 1),x(t - 2), \ldots ,x(t - d))$$

where $X_{t}$ signifies the forecasting results from the NARANN method based on the previous given values at lagged period *d*.

In this study, the modeling procedures consist of three steps. First, the whole data were divided into two blocks including training samples (from 28 February 2020 to 15 June 2020) and testing samples (from 16 June 2020 to 27 June 2020). To develop an effective and accurate NARANN model, the effective training samples were further partitioned into training (80% of the training samples), validation (10%), and testing (10%) subseries by use of the *dividerand* function in MATLAB software. Second, the number of hidden neurons and delays *d* were investigated by trial and error by use of the Levenberg–Marquardt algorithm in an open feedback form^33^. Whilst the response plot between the estimated outputs and targets, the ACF plot, along with the mean square error (MSE) and correlation coefficient (R) were computed until the best possible specification was determined^53^. Finally, the training open-loop form was closed to make a multi-step-ahead forecast.

### A hybrid model of EEMD-ARIMA-NARANN

#### EEMD

Although the EMD method has been widely employed to deal with the noisy nonlinear and non-stationary processes in signal analysis, it has been shown that this method suffers from two major shortcomings, including the edge-effects and mode-mixing in applications^39,54,55^, particularly for the mode-mixing issue, it can not only lead to the mixing of different scale vibration modes but also even result in the loss of the physical meaning of the decomposed IMFs terms^40^. To compensate for the weaknesses of the EMD method, an advanced EEMD technique was therefore introduced based on the EMD method^39^. This EEMD technique resolved the mode-mixing issue by defining the original each IMFs term as the average of an ensemble of experiments, and each IMFs term consists of the signal and noise of finite-amplitude^54^. The decomposition processes of the EEMD approach can be done as below:

Firstly, adding a white noise series $w(t)$ to the original series $x(t)$, and then the produced new time series was defined as

2 $$Y(t) = x(t) + w(t)$$

Secondly, decomposing this new time series into the IMFs terms by use of the EMD method.

Thirdly, repeating the first and second steps using different white noise series, and the obtained results were added to the original time series each time.

Finally, averaging the ensemble of the IMFs terms from the EMD method.

At the decomposition stage, determining the number of the ensembles and the amplitudes of the added white noise series is very crucial for the resultant results^43^. Fortunately, these two parameters can be determined by use of a well-demonstrated statistical rule^39^

3 $$\varepsilon_{n} = \frac{\varepsilon }{N}$$

where N is the number of the ensembles, $\varepsilon$ represents the amplitudes of the added white noise series, and $\varepsilon_{n}$ refers to the standard error. It has been shown that the EEMD technique can obtain a satisfactory result when the ensemble numbers were 100 and the amplitudes of added white noise series were 0.2 times standard deviation^39,56^.

##### EEMD-ARIMA-NARANN mixture model

**Figure 1:**
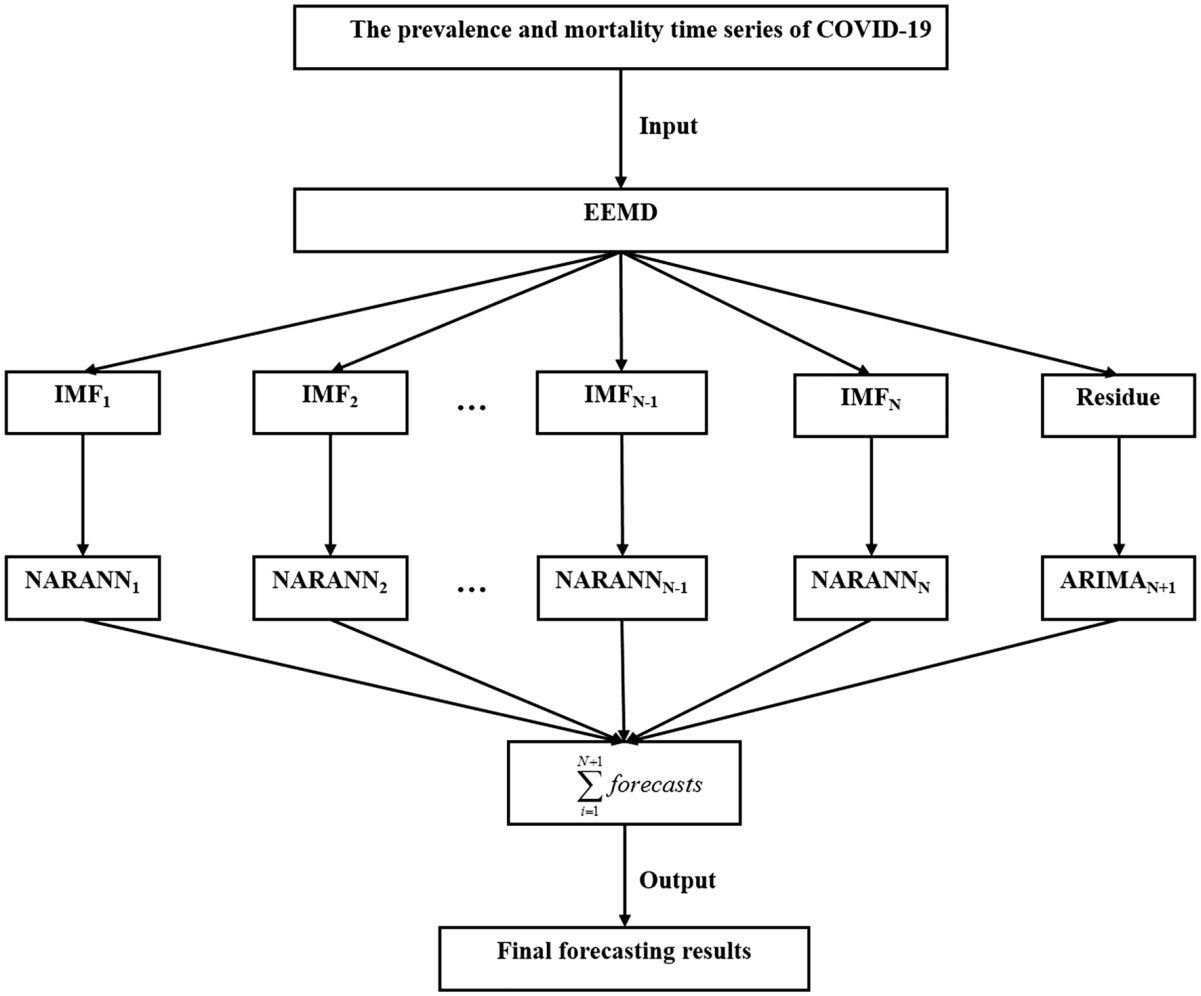
Flow chart of the novel data-driven EEMD-ARIMA-NARANN mixture method.

To achieve the goal of making full use of the constituent linear and nonlinear components in the object series, inspired by the “decomposition and ensemble” idea of the EEMD method and its powerful flexible nonlinear mapping capacity of the NARANN method^57^, the EEMD-ARIMA-NARANN mixture method was thus constructed. In this advanced mixture model-developing process, the prevalence and mortality time series of COVID-19 were first decomposed into various IMFs and residue terms. Then, each of IMFs terms was modeled by use of an adequate NARANN method; whereas the residue term was modeled by use of an adequate ARIMA method. Finally, the results from our proposed mixture method could be obtained by combing the forecasts from the ARIMA and NARANN models (Fig. 1). By doing so, the new data-driven mixture technique can capture both linear and nonlinear patterns simultaneously in the prevalence and mortality series of COVID-19. The specific representation of our proposed EEMD-ARIMA-NARANN mixture method can be expressed as

4 $$\hat{b}_{t} = \sum\limits_{i = 1}^{N} ( f(IMF_{1} (t - 1), \ldots ,IMF_{1} (t - d)) + \cdots + (f(IMF_{N} (t - 1), \ldots ,IMF_{N} (t - d))$$

5 $$\hat{y} = {\hat{\text{a}}}_{{\text{t}}} + \hat{b}_{t}$$

where $\hat{y}$ refers to the estimated results from the EEMD-ARIMA-NARANN mixture technique, ${\hat{\text{a}}}_{{\text{t}}}$ represents the estimated results from the ARIMA model, $\hat{b}_{t}$ is the estimated results from the NARANN model.

##### Assessing model performance

In this study, four statistical measures of error, including root mean square percentage error (RMSPE), mean absolute deviation (MAD), mean error rate (MER), and mean absolute percentage error (MAPE), were calculated to evaluate the accuracy of forecasts. The above statistical measures of error had smaller values, indicating a better model.

6 $${\text{RMSPE}} = \sqrt {\frac{1}{N}\sum\limits_{i = 1}^{N} {\left( ({X_{i} - \overline{X}_{i})/{X_{i}} } \right)^{{2}} } }$$

7 $${\text{MAD}} = \frac{1}{N}\sum\limits_{i = 1}^{N} {\left| {X_{i} - \hat{X}_{i} } \right|}$$

8 $${\text{MER}} = \frac{{\frac{1}{N}\sum\limits_{i = 1}^{N} {\left| {X_{i} - \hat{X}_{i} } \right|} }}{{\overline{X}_{i} }}$$

9 $${\text{MAPE}} = \frac{1}{N}\sum\limits_{i = 1}^{N} {\frac{{\left| {X_{i} } \right. - \left. {\hat{X}_{i} } \right|}}{{X_{i} }} \times 100}$$

here $X_{i}$ signifies the prevalence and mortality data of COVID-19, $\hat{X}_{i}$ is the estimates using the chosen approaches, $\overline{X}_{i}$ refers to the mean of the prevalence and mortality data of COVID-19, and $N$ stands for the number of simulations and forecasts.

## Results

### Development of the ARIMA model

**Figure 2:**
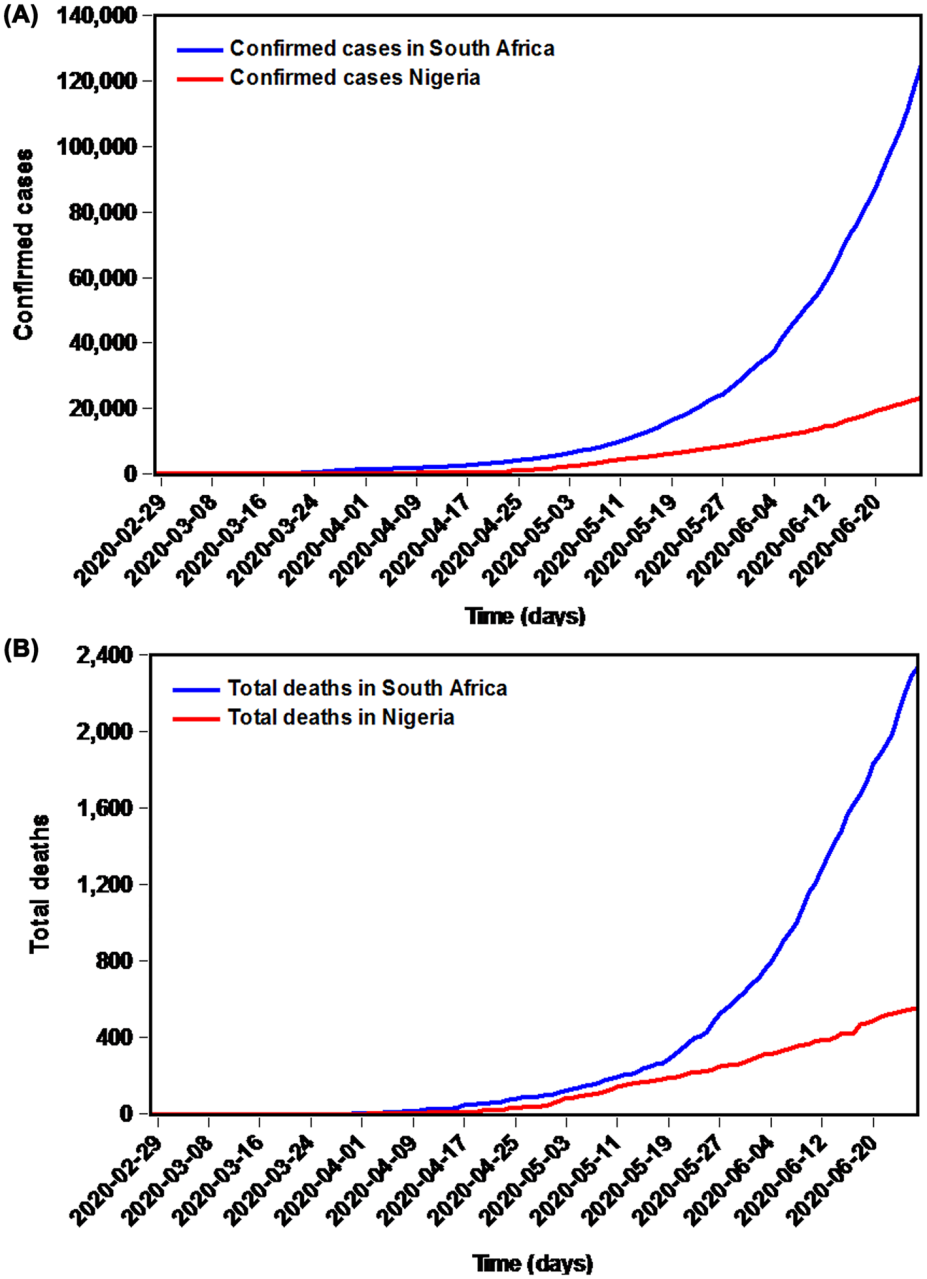
Time series plots showing the prevalence and mortality data of COVID-19 in South Africa and Nigeria (**A**) The overall confirmed cases in these two countries; (**B**) The overall deaths in these two countries.

**Figure 3:**
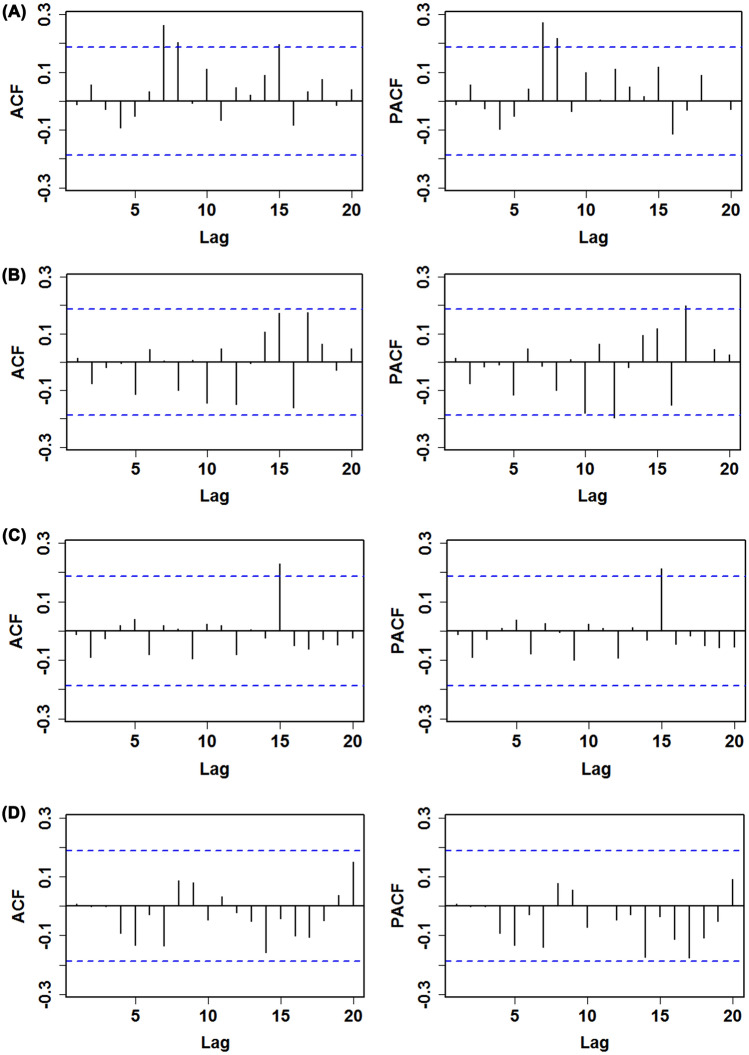
Autocorrelogram (ACF) and partial autocorrelogram (PACF) of the residuals generated by the best ARIMA model. (**A**) Sample ACFs and PACFs of the residuals for the prevalence dada in South Africa; (**B**) Sample ACFs and PACFs of the residuals for the mortality dada in South Africa; (**C**) Sample ACFs and PACFs of the residuals for the prevalence dada in Nigeria; (**D**) Sample ACFs and PACFs of the residuals for the mortality dada in Nigeria. As shown, almost all sample ACFs and PACFs fell within the estimated 95% uncertainty levels across different lags except for the sample ACFs at lags 7, 8, and 15, along with the sample PACFs at lags 7 and 8 in (**A),** the sample PACFs at lags 12 and 17 in (**B),** and the sample ACFs and PACFs lag 15 in (**C)** (which are also reasonable because some higher-order correlation coefficients readily exceed the estimated 95% uncertainty levels by chance). These results meant that the residuals from identified ARIMA models for different datasets were without pattern, suggesting that the selected ARIMA models appear to be suitable for capturing the dynamic dependency structure in the object series.

During the study span, the overall confirmed cases totaled 12,459 in South Africa and 23,298 in Nigeria, with a daily mean of 1030 and 193 cases, respectively. Out of them, there were overall 2340 deaths in South Africa and 554 deaths in Nigeria, with a daily mean of 20 and 5 cases, respectively. As shown in Fig. 2, the prevalence and mortality time series displayed an apparent increasing trend, so the differencing is required to remove the trend effects of these target series. After differencing, an ADF test was employed to the differenced series, and the resulting statistics for the differenced series are illustrated in Table S1, indicating a stationary series. Thus, the possible values of the ARIMA models’ key parameters were crudely determined based on these stationary series. As illustrated in Table 1, it appeared that the sparse coefficient ARIMA (2, 2, (1, 3)) (AIC = 1482.590, CAIC = 1483.441, BIC = 1498.642, and Log-likelihood = -736.290) and ARIMA (0, 2,(1, 3, 4)) (AIC = 733.390, CAIC = 733.980, BIC = 746.750, and Log-likelihood = − 362.690) specifications were expected to be considered the best models for simulating the prevalence and mortality data, respectively, in South Africa because the measurement metrics of AIC, CAIC, and BIC provided the lowest values, and log-likelihood gave the greatest value among all the possible models. Furthermore, as illustrated in Tables 2 and 3, Fig. 3, the identified key parameters of the best-fitting ARIMA models showed a statistical significance (*p* < 0.05) and the Box-Ljung Q tests for the error series from these best models suggested no statistical significance at different lags (*p* > 0.05), these results meant that the identified optimal ARIMA models are adequate for modeling the target data. Similarly, the diagnostic checking for the best ARIMA models could be done on the residuals from the prevalence and mortality data in Nigeria (Tables 1, 2, 3 and Fig. 3), it was demonstrated that the ARIMA (1, 2, 2) and sparse coefficient ARIMA (0, 2,(1, 2, 4)) models were also suitable for modeling the prevalence and mortality data, respectively, in Nigeria. Accordingly, these preferred ARIMA models determined can be used to forecast the epidemics in the next days.

**Table 1:** The possible tested ARIMA models for the prevalence and mortality time series of COVID-19 in South Africa and Nigeria.

Country	Model	AIC	CAIC	BIC	Log likelihood
South Africa	Prevalence data				
	ARIMA (2, 2, (1, 3))	1482.590	1483.441	1498.642	− 736.290
	ARIMA (0, 2, 1)	1485.030	1485.140	1490.370	− 740.510
	ARIMA (0, 2, 2)	1486.980	1487.210	1495.000	− 740.490
	ARIMA (0, 2, 3)	1488.880	1489.280	1499.580	− 740.440
	ARIMA (1, 2, 3)	1484.830	1485.420	1498.190	− 737.420
	ARIMA (2, 2, 0)	1487.060	1487.300	1495.080	− 740.530
	Mortality data				
	ARIMA (0, 2, (1, 3, 4))	733.390	733.980	746.750	− 362.690
	ARIMA (0, 2, 1)	750.650	750.770	756.000	− 373.330
	ARIMA (0, 2, 2)	752.540	752.780	760.560	− 373.270
	ARIMA (0, 2, 3)	742.200	742.590	752.890	− 367.100
	ARIMA (1, 2, 1)	752.610	752.840	760.630	− 373.310
Nigeria	Prevalence data				
	ARIMA (1, 2, 2)	1319.300	1319.690	1329.990	− 655.650
	ARIMA (0, 2, 1)	1323.680	1323.790	1329.020	− 659.840
	ARIMA (0, 2, 2)	1322.700	1322.930	1330.720	− 658.350
	ARIMA (1, 2, 0)	1349.550	1349.660	1354.890	− 672.770
	ARIMA (1, 2, 1)	1324.100	1324.330	1332.120	− 659.050
	ARIMA (2, 2, 1)	1322.600	1323.000	1333.300	− 657.300
	Mortality data				
	ARIMA (0, 2, (1, 2, 4))	591.220	591.810	604.580	− 291.610
	ARIMA (0, 2, 1)	600.390	600.510	605.740	− 298.200
	ARIMA (0, 2, 2)	601.940	602.170	609.960	− 297.960
	ARIMA (0, 2, 3)	602.470	602.860	613.160	− 297.240
	ARIMA (0, 2, 4)*	592.980	593.570	606.340	− 291.490

*ARIMA* Autoregressive integrated moving average method, *AIC* Akaike information criteria, *CAIC* consistent AIC, *BIC* Bayesian information criterion.

*Represents the parameters were no significantly statistical difference.

**Table 2:** The identified parameters of the best-fitting ARIMA models for the prevalence and mortality time series of COVID-19 in South Africa and Nigeria.

Country	Model	Parameters	Estimates	Standard error	t	*p*
South Africa	Prevalence data					
	ARIMA (2, 2, (1, 3))	AR1	− 1.390	0.146	− 9.521	< 0.001
		AR2	− 0.432	0.141	− 3.064	0.001
		MA1	1.257	0.093	13.516	< 0.001
		MA3	− 0.367	0.086	− 4.267	0.001
	Mortality data					
	ARIMA (0, 2, (1, 3, 4))	MA1	− 0.958	0.084	− 11.405	< 0.001
		MA3	0.344	0.134	2.567	0.006
		MA4	0.362	0.099	3.657	< 0.001
Nigeria	Prevalence data					
	ARIMA (1, 2, 2)	AR1	0.632	0.201	3.144	0.001
		MA1	− 1.646	0.153	− 10.758	< 0.001
		MA2	0.755	0.120	6.292	< 0.001
	Mortality data					
	ARIMA (0, 2, (1, 2, 4))	MA1	− 0.928	0.097	− 9.567	< 0.001
		MA2	− 0.198	0.119	− 1.664	0.0495
		MA4	0.437	0.069	6.333	< 0.001

*ARIMA* Autoregressive integrated moving average method, *AR* autoregressive method, *MA* moving average method.

**Table 3:** Box-Ljung Q test for the residual series from the best ARIMA and NARANN models.

Lags	Prevalence data				Mortality data			
	ARIMA		NARANN		ARIMA		NARANN	
	Box-Ljung Q	*p*	Box-Ljung Q	*p*	Box-Ljung Q	*p*	Box-Ljung Q	*p*
1	0.019	0.890	0.019	0.890	0.022	0.881	0.004	0.948
2	0.388	0.824	0.954	0.621	0.677	0.713	0.005	0.998
3	0.481	0.923	1.038	0.792	0.721	0.868	0.005	1.000
4	1.473	0.832	1.082	0.897	0.724	0.948	1.010	0.908
5	1.796	0.877	1.278	0.937	2.229	0.817	3.096	0.685
6	1.925	0.927	2.061	0.914	2.474	0.871	3.193	0.784
7	10.181	0.179	2.105	0.954	2.477	0.929	5.348	0.618
8	15.118	0.057	2.111	0.977	3.659	0.887	6.263	0.618
9	15.126	0.088	3.228	0.955	3.663	0.932	7.036	0.633
10	16.616	0.083	3.292	0.974	6.233	0.795	7.296	0.697
11	17.150	0.104	3.336	0.986	6.513	0.837	7.422	0.764
12	17.424	0.134	4.141	0.981	9.342	0.674	7.478	0.825

*ARIMA* Autoregressive integrated moving average method, *NARANN* nonlinear autoregressive artificial neural network.

### Construction of the NARANN model

**Figure 4:**
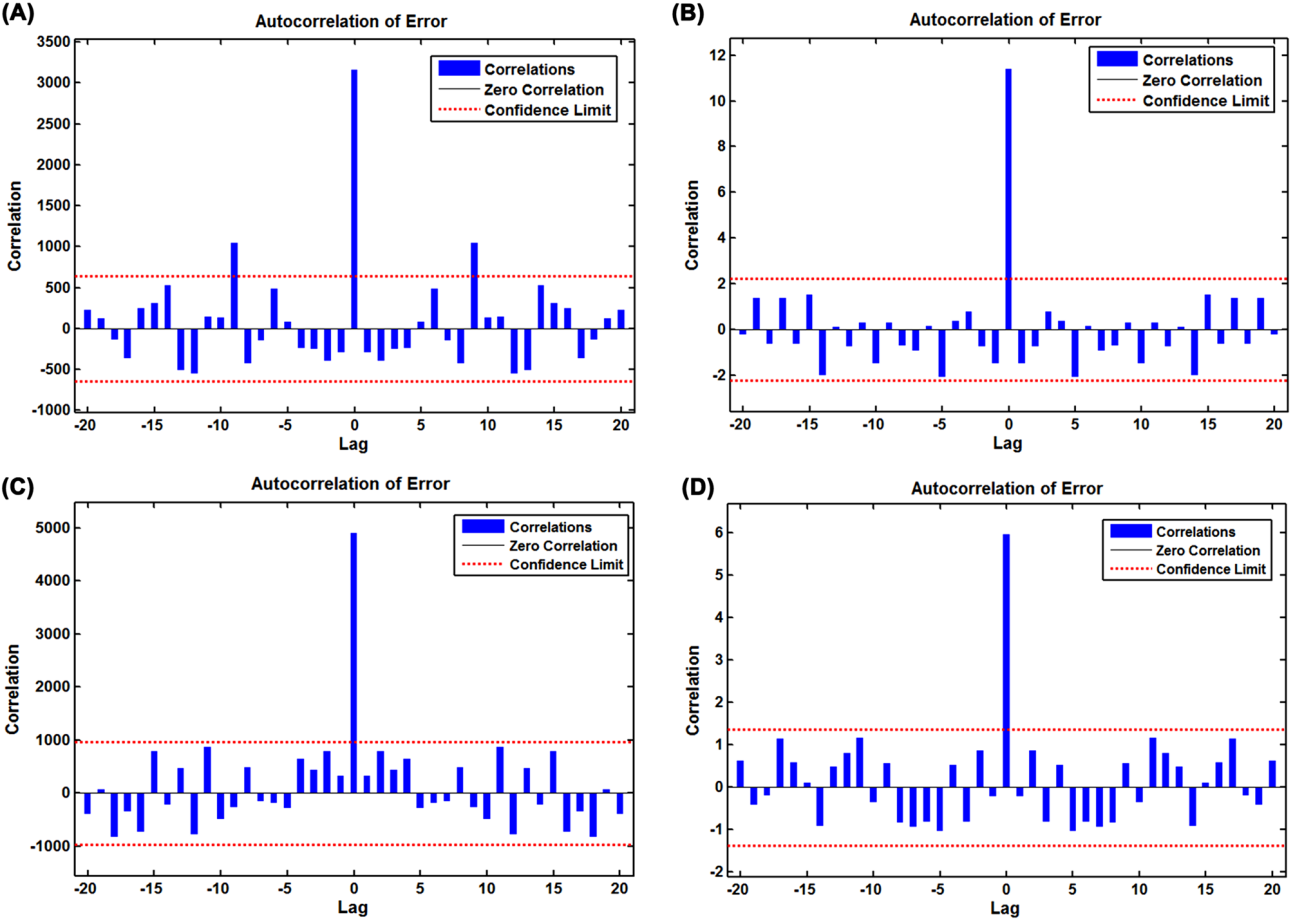
Autocorrelogram (ACF) of the residuals generated by the best NARANN model. (**A**) Sample ACFs of the residuals for the prevalence dada in South Africa; (**B**) Sample ACFs of the residuals for the mortality dada in South Africa; (**C**) Sample ACFs of the residuals for the prevalence dada in Nigeria; (**D**) Sample ACFs of the residuals for the mortality dada in Nigeria.

**Figure 5:**
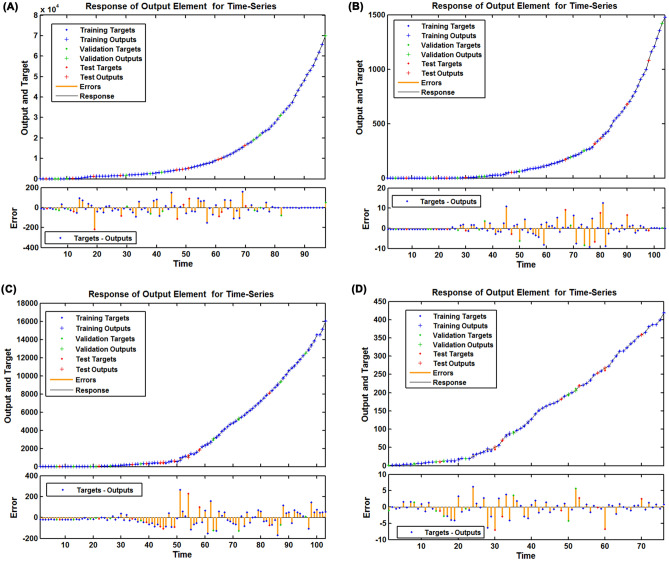
Time series displaying the response results between inputs and outputs. (**A**) Response plot between inputs and outputs for the prevalence dada in South Africa; (**B**) Response plot between inputs and outputs for the mortality dada in South Africa; (**C**) Response plot between inputs and outputs for the prevalence dada in Nigeria; (**D**) Response plot between inputs and outputs for the mortality dada in Nigeria. These plots display which samples were treated as the training, validation and testing datasets, and illustrate the corresponding errors between inputs and targets. It could be seen that the vast majority of data points had smaller errors between inputs and targets, indicating that the identified NARANN methods seem to be adequate for estimating the epidemiological trends of COVID-19 in the study regions.

To obtain the preferred NARANN model, the different number of hidden units ranging from 1 to 20 and feedback delays ranging from 1 to 6 were trained by trial and error. After trying, it was found that the NARANN with 15 hidden units and 6 delays and the NARANN with 14 hidden units and 5 delays tended to be identified as the optimal specifications for mimicking the prevalence and mortality data, respectively, in South Africa as the NARANN (15,6) and NARANN (14,5) specifications showed the lowest MSE values in the training (2648.213 and 9.710, respectively), validation (1595.504 and 12.849, respectively), and testing (8647.196 and 24.024, respectively) subsets, along with the greatest R values in the training (1 and 1, respectively), validation (1 and 1, respectively), and testing (1 and 1, respectively) subsets of the prevalence and mortality data among all the potential models (Tables 3 and 4, Figures S1 and S2). Moreover, almost all autocorrelation coefficients of the resulting errors fell into the estimated 95% uncertainty level (UL) at different lags and the response plots between inputs and outputs showed that the resulting residuals presented an acceptable level of fluctuation in their corresponding subsets (Figs. 4, 5). The above-mentioned results intimated that the identified two best NARANN specifications offered reliable estimates for the prevalence and mortality series in South Africa. Likewise, we determined the best NARANN (15,6) and NARANN (14,6) specifications for fitting the prevalence and mortality data, respectively, in Nigeria according to the modeling steps, and the statistical checking results exhibited that these identified NARANN specifications were also appropriate (Tables 3, 4, Figs. 4, 5, S3 and S4). Therefore, these resulting best NARANN models can be applied to the target series to generate forecasts for the testing samples.

**Table 4:** The identified parameters of the best NARANN and EEMD-ARIMA-NARANN hybrid models for different target series.

Country	Target series	Hidden units	Delays	MSE			R			
				Training subseries	Validation subseries	Testing subseries	Training subseries	Validation subseries	Testing subseries	Overall
South Africa	Prevalence data									
	Original series	15	6	2648.213	1595.504	8647.196	1.000	1.000	1.000	1.000
	IMF1	15	5	50,572.372	84,373.288	136,600.627	0.864	0.742	0.632	0.834
	IMF2	16	4	8177.455	3132.033	8164.032	0.959	0.969	0.944	0.960
	IMF3	15	5	80.456	96.150	439.063	1.000	0.999	0.998	1.000
	IMF4	14	4	5.420	1.215	4.841	1.000	1.000	1.000	1.000
	IMF5	14	5	6.780	4.005	3.993	1.000	1.000	1.000	1.000
	Mortality data									
	Original series	14	5	9.710	12.849	24.024	1.000	1.000	1.000	1.000
	IMF1	17	5	34.933	99.083	32.655	0.823	0.626	0.920	0.789
	IMF2	16	5	2.428	7.065	5.659	0.976	0.922	0.981	0.971
	IMF3	14	5	4.363	1.155	1.411	0.999	0.999	0.999	0.999
	IMF4	15	4	2.444	2.518	6.129	1.000	1.000	1.000	1.000
	IMF5	15	4	1.672	4.768	4.259	1.000	1.000	1.000	1.000
Nigeria	Prevalence data									
	Original series	15	6	4417.994	3853.800	9902.660	1.000	1.000	0.999	1.000
	IMF1	17	5	5061.116	8908.892	15,396.154	0.816	0.764	0.575	0.763
	IMF2	16	5	774.071	1073.004	494.054	0.933	0.930	0.981	0.938
	IMF3	15	5	9.138	27.153	18.503	0.999	0.991	0.990	0.998
	IMF4	16	6	1.751	2.290	7.026	1.000	1.000	1.000	1.000
	IMF5	15	5	1.632	1.278	1.000	1.000	1.000	1.000	1.000
	Mortality data									
	Original series	14	6	4.259	9.533	15.204	1.000	0.999	1.000	1.000
	IMF1	18	5	2.467	6.268	7.791	0.890	0.779	0.235	0.830
	IMF2	16	5	3.259	4.492	8.789	0.978	0.970	0.979	0.976
	IMF3	15	5	3.690	2.591	4.244	1.000	1.000	0.997	0.999
	IMF4	14	6	1.292	2.924	2.852	1.000	1.000	1.000	1.000
	IMF5	15	5	2.628	4.384	5.654	1.000	1.000	1.000	1.000

*MSE* mean square error.

### Establishment of the EEMD-ARIMA-NARANN hybrid model

**Figure 6:**
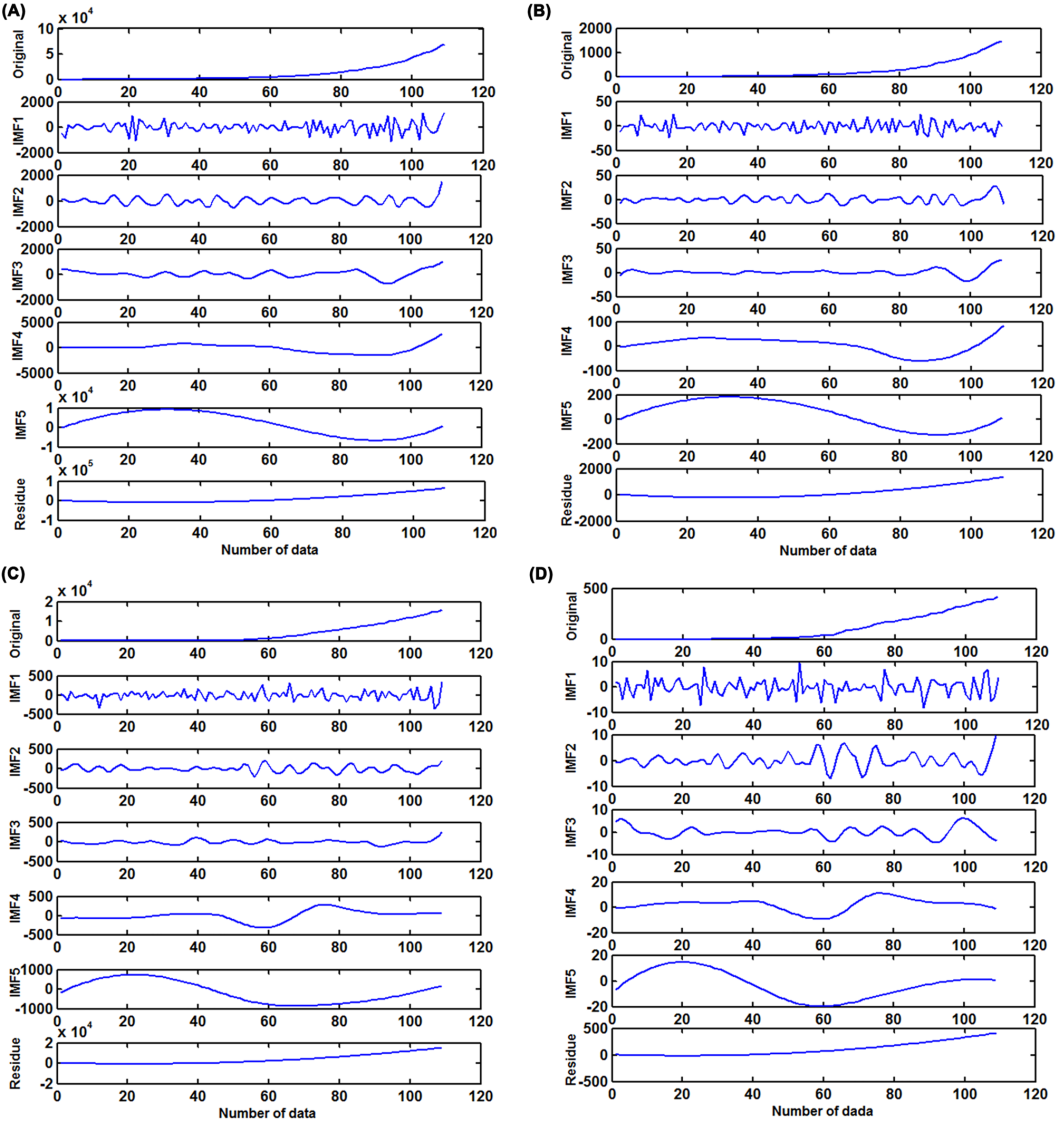
Intrinsic Mode Functions (IMFs) subseries via decomposing the original prevalence and mortality time series. (**A**) The resulting IMFs subseries by decomposing the prevalence series in South Africa; (**B**) The resulting IMFs subseries by decomposing the mortality series in South Africa; (**C**) The resulting IMFs subseries by decomposing the prevalence series in Nigeria; (**D**) The resulting IMFs subseries by decomposing the mortality series in Nigeria.

Based on the decomposed procedures, the original target series was decomposed into different IMFs and residues (Fig. 6). Subsequently, the residues representing the trends of the target series were used to establish the ARIMA model, and the obtained best-fitting ARIMA models and their goodness of fit statistics for different target series are listed in Table 5; whereas the IMFs components representing the detailed (nonlinear) information contained in the target series were employed to develop the NARANN models, and the identified best-fitting NARANN models and their diagnostic testing results for various IMFs series are summarized in Table 4. Then each decomposed series is fitted and predicted by adopting the most appropriate target models and the resulting in-sample simulations and out-of-sample forecasts can be summed to obtain the final results from the advanced EEMD-ARIMA-NARANN hybrid model.

**Table 5:** The identified parameters of the best-fitting ARIMA models for the decomposed residue of the COVID-19 the prevalence and mortality in South Africa and Nigeria.

Country	Model	Parameters	Estimates	Standard Error	t	*p*	R^2^	Stationary R^2^	Normalized BIC	Box-Ljung Q	
										Statistics	*p*
South Africa	Residue of the prevalence data										
	ARIMA(0, 4, 2)	MA1	− 1.019	0.084	− 12.164	< 0.001	1.000	0.493	− 13.179	5.616	0.992
		MA2	− 0.536	0.084	− 6.401	< 0.001					
	Residue of the mortality data										
	ARIMA(1, 3, 0)	AR1	1.000	2.14E− 05	46,715.700	< 0.001	1.000	–	− 21.213	0.004	1.000
Nigeria	Residue of the prevalence data										
	ARIMA(0, 4, 2)	MA1	− 0.803	0.097	− 8.293	< 0.001	1.000	0.382	− 16.364	3.248	1.000
		MA2	− 0.194	0.097	− 1.995	0.049					
	Residue of the mortality data										
	ARIMA(1, 4, 0)	AR1	0.662	0.073	9.083	< 0.001	1.000	0.426	− 24.307	8.47	0.955

*ARIMA* Autoregressive integrated moving average method, *BIC* Bayesian information criterion, *AR* autoregressive method, *MA* moving average method.

### Comparisons of forecasting accuracy level between models

**Figure 7:**
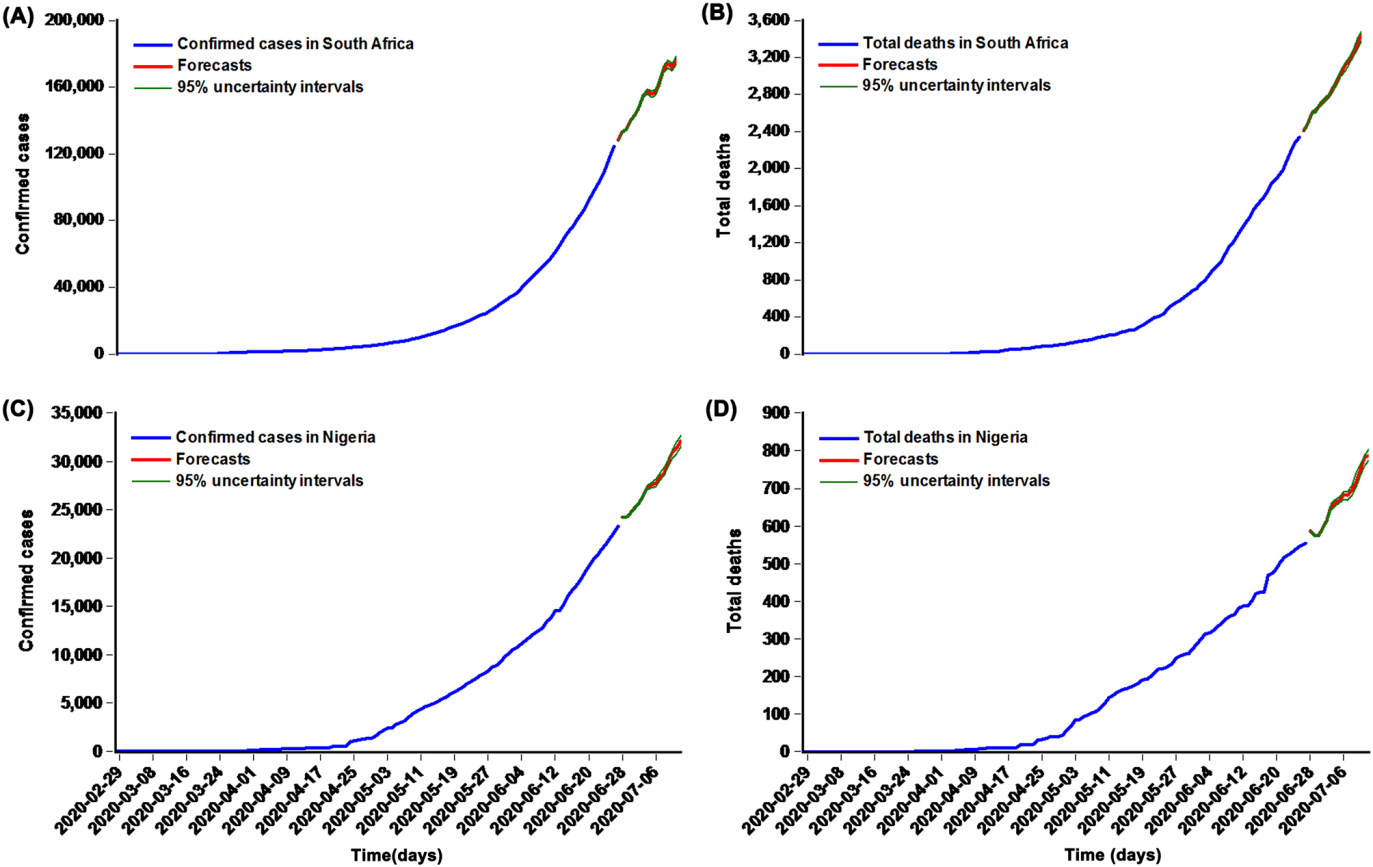
The next 15-day forecasts and their 95% uncertainty levels for the prevalence and mortality data using the best-fitting EEMD-ARIMA-NARANN mixture model. (**A**) The next 15-day forecasts for the prevalence data in South Africa; (**B**) The next 15-day forecasts for the mortality data in South Africa; (**C**) The next 15-day forecasts for the prevalence data in Nigeria; (**D**) The next 15-day forecasts for the mortality data in Nigeria.

We discovered that the EEMD-ARIMA-NARANN mixture model showed the lowest values of the measurement metrics, including MAD, MAPE, MER, and RMSPE, in addition to the RMSPE value in the prevalence data of Nigeria by comparing the forecasts for the testing samples from the selected best-fitting three models in the study regions (Table 6). Consequently, we can conclude that our proposed mixture model is superior to the basic ARIMA and NARANN models. Further, we re-established our proposed hybrid model to forecast the future 15-day epidemiological trends of the COVID-19 prevalence and mortality based on the overall data, and the resulting best models and the final forecasts are visible in Figs. 7, S5 and S6, Tables S1–S5. So the next 15-day forecasts of confirmed cases may be 176,570 (95% UL 173,607 to 178,476) in South Africa and 32,136 (95% UL 31,568 to 32,641) in Nigeria, and the forecasts of total deaths may be 3454 (95% UL 3384 to 3487) in South Africa and 788 (95% UL 775 to 804) in Nigeria (Table S5).

**Table 6:** Comparisons of the predictive abilities for the testing samples of the prevalence and mortality time series of COVID-19 among these three selected models in South Africa and Nigeria.

Country	Model	Predictive performance for prevalence data				Predictive performance for mortality data			
		MAD	MAPE	MER	RMSPE	MAD	MAPE	MER	RMSPE
South Africa	ARIMA	2064.810	1.966	0.021	0.026	67.923	3.213	0.035	0.041
	NARANN	2996.471	2.908	0.031	0.036	58.052	3.102	0.030	0.037
	Hybrid model	1261.426	1.170	0.013	0.020	47.119	2.435	0.024	0.030
Reduced percentages (%)									
C vs. A		38.908	40.488	38.095	23.077	30.629	24.214	31.429	26.829
C vs. B		57.903	59.766	58.065	44.444	18.833	21.502	20.000	18.919
Nigeria	ARIMA	326.775	1.620	0.016	0.017	27.959	5.477	0.056	0.058
	NARANN	371.259	1.792	0.019	0.028	20.913	4.118	0.041	0.057
	Hybrid model	280.279	1.388	0.014	0.017	14.490	2.917	0.029	0.032
Reduced percentages (%)									
C vs. A		14.229	14.321	12.500	0.000	48.174	46.741	48.214	44.828
C vs. B		24.506	22.545	26.316	39.286	30.713	29.165	29.268	43.860

*ARIMA* Autoregressive integrated moving average method, *NARANN* nonlinear autoregressive artificial neural network, *MAD* Mean absolute deviation, *MAPE* Mean absolute percentage error, *MER* Mean error rate, *RMSPE* Root mean square percentage error, A refers to the ARIMA model, B refers to the NARANN model, C refers to the EEMD-ARIMA-NARANN hybrid model.

## Discussion

Effective prevention and control plans are needed to curb and harness the rapid transmission of the COVID-19 outbreak. Early nowcasting and forecasting are essential to forming such plans as the allocation of limited health resources, the timely adjustment of the current intervention strategies, the arrangement of production activities, and even the local economic development^30,31,58^. For this reason, it is imperative to develop statistical techniques with high forecasting accuracy and reliability. Time series modeling is a useful aid for developing underlying hypotheses to analyze the current epidemic patterns and to predict the spreading dynamics of different diseases in the near future^4,7^. As far as we are aware, this is the only study to analyze and forecast the epidemiological trends of the COVID-19 prevalence and mortality time series in South Africa and Nigeria by use of a novel data-driven EEMD-ARIMA-NARANN hybrid technique, and a series of modeling experiments indicated that this new hybrid technique produced lower forecasting errors over the basic ARIMA and NARANN methods by comparing the measurement metrics, such as MAD, MAPE, MER, and RMSPE (Table 6). These results meant our proposed hybrid method has a greater potential to track the dynamic dependence characteristics during the epidemic process of COVID-19 relative to the others used in this study, which may act as a profitable tool-supportive for policymakers to develop appropriate prevention and control strategies and measures in both mitigating the outbreak and reducing the deaths due to COVID-19 pandemic. Whilst this hybrid model is also of great value in assessing the effects of the current public interventions. For example, if this model forecasted a remarkably higher epidemic level than the actual in the coming periods, suggesting that the current measures could take effect in the target population; otherwise, indicating that the current public interventions could be required to be reinforced or additional plans could be needed. In addition, the basic ARIMA and NARANN models also provided a high forecasting accuracy for our target data in light of the above four measurement metrics.

The most versatile method to fit the time series data is the ARIMA model, which postulates that there is a certain linear association between the future epidemics of a given series and the past and present states of the target series, and thus this model can not only be used to model nonseasonal data but also seasonal data, and such benefit as nonstationary data^48,49^. Yet for nonstationary series, it requires to be differenced and/or transformed with logarithm or square root^50^. For instance, Yousaf et al. built the ARIMA (0, 2, 1), ARIMA (2, 2, 0), and ARIMA (1, 2, 1) models to study and predict the accumulative confirmed cases, recoveries, and deaths of COVID-19, respectively, for the upcoming month in Pakistan^19^. Ceylan established the ARIMA (0, 2, 1), ARIMA (1, 2, 0), and ARIMA (0, 2, 1) models to forecast the total reported cases of COVID-19 in Italy, Spain, and France, respectively^7^. Even though these obtained ARIMA models have high forecasting accuracy and reliability, the major disadvantage of the ARIMA model is its linear assumption, which makes it difficult to handle the randomness in the target series^52^. Hence, we proposed a novel data-driven EEMD-ARIMA-NARANN hybrid model to overcome the limitation of the basic model. It can be said that this data-driven mixture technique shows a strong capacity to improve the forecasting power for the prevalence and mortality data of COVID-19 in that the principal advantage of such a model facilitates to identify the preferred hybridization by decomposing the target data into various multi-scale levels to consider the underlying trend and random parts simultaneously by use of the different types of models. Given the forecasting superiority of our proposed data-driven hybrid method, it seems that this hybrid model is also useful in nowcasting and forecasting the epidemiological trends of the COVID-19 prevalence and mortality time series in other regions or other infectious diseases^44^. Of note, current studies found that some other forecasting tools (e.g., the new innovations state space modeling framework^59^, long short-term memory neural network^60^, advanced error-trend-seasonal (ETS) framework^61^, α-Sutte Indicator^62^, and SBDiEM^30^) performed a highly accurate forecast for the epidemiological trends of COVID-19. As a result, to further our research we are planning to make a comparative study between our proposed EEMD-SARIMA-NARANN hybrid model and the ones above. The contributions of the current work are several-fold. First, at least 14.321% and at most 40.488%, along with at least 22.545% and at most 59.766% of computational accuracies are achieved compared with the ARIMA and NARANN models, respectively, when using the MAPE (which is the most frequently used index to judge the predictive performance) to measure the forecasting accuracy. Second, this work presents a new data-driven integrated system in a more reasonable way compared with the conventional mixture pattern. Third, this new data-driven hybrid model may be generalized to estimate the epidemic patterns in other regions seriously affected by the COVID-19 outbreak.

Given the outbreak trends of COVID-19 and the situation of the health infrastructure and services in Africa, there is a great concern on whether African regions’ health system capacity is able to duly and effectively meet the requirements of the medical supplies for the increased confirmed cases. For this reason, we used our proposed mixture technique to predict the next 15-day confirmed cases and deaths in South Africa and Nigeria. Particularly in South Africa, the infected individuals show an exponential trend since 18 May 2020 (Figs. 2, 7), and even worse, our prediction results display that the epidemiological trends of the outbreak may still be rapidly increasing with an average of around 3465 confirmed cases and 75 deaths per day in the upcoming 15 days in South Africa (Fig. 7A,B, Table S5), and it needs more time to reach the platform in the morbidity. Therefore, more strict or additional precautionary measures are required to reduce the rapid spreading of COVID-19 (e.g., increasing the number of doctors, pharmacists, medical students, and other health workers who can offer their expertise in the frontlines of the pandemic response, strengthening the overnight curfew management to prevent the social interaction, raising public awareness by strengthening advocacy, issuing more stringent lockdown rules, building more mobile cabin hospitals to treat the mild patients, forcing mandated face-covering in public, suspending trans-regional public transportation, suspending or prohibiting tourism across regions, strengthening inspection and quarantine, extending the closure period of public places such as schools, universities and church, supporting the home office work, prohibiting possible social gatherings, accelerating research on the vaccines and clinical treatment programmes, and seeking help from other countries in a position to do so)^12,19,31,60,63^. Nigeria that was hit the second hardest with the COVID-19 outbreak is witnessing a downward trend in the COVID-19 prevalence and mortality with daily 590 estimated confirmed cases and 16 deaths in the next 15 days (Fig. 7C,D, Table S5). However, strict prophylactic measures still need to be implemented in Nigeria to avoid the rebounding of the outbreak.

The findings in this report are subject to some shortcomings. Firstly, accurate statistics on the prevalence and mortality data in these two study regions are vital for the understanding of the epidemic patterns of COVID-19 by use of our proposed data-driven EEMD-ARIMA-NARANN hybrid technique. However, the limited nuclear acid detection ability may result in under-diagnosis or under-reporting for the prevalence and mortality data during the COVID-19 outbreak. Secondly, in the NARANN method-developing process, there is currently a lack of general guidelines for selecting the number of hidden neurons and delays. In applications, repeated training is required. Thirdly, although this data-driven mixture technique does a good job of estimating the epidemic patterns of COVID-19 in this study, whether this data-driven mixture technique can perform a highly accurate prediction for the epidemiological trends of COVID-19 in other regions or other infectious contagious diseases, more work will need to be done. Fourthly, the forecasting performance under the EEMD-ARIMA-NARANN hybrid technique may be further improved by integrating some related factors (e.g., internet search queries, meteorological parameters, air pollution indicators, and policy intervention), and further studies, which take these factors related to the COVID-19 into account, will be very interesting. However, this failed to be investigated in the current work. Lastly, the forecasting reliability level of this data-driven mixture technique may decrease with the increase of the forecasting periods. Therefore, the new real-time data should be integrated into the model to ensure its forecasting accuracy.

## Conclusions

Insights from the time series modeling are extremely invaluable for the policymaker to plan effective prevention and control strategies in order to make the outbreak under control well in the future. In this work, we proposed a new data-driven EEMD-ARIMA-NARANN mixture technique, and it is demonstrated that the predicted values from this mixture model show better consistency with the actual observations than the basic ARIMA and NARANN methods, which can function as a helpful policy-supportive tool to plan and prepare medical supplies effectively, and thus favoring to alleviate the outbreak in South Africa and Nigeria over the upcoming days or weeks. It is significant to stress that the estimated values may differ from the observed values looking at the strategic preparedness and the measures taken by the government of these study regions. Also, our proposed hybrid model may be of great help to estimate and forecast the future epidemic trends in other regions severely affected by this crisis.

## Supplementary Information

Supplementary Information.

## Data Availability

All the data can be obtained from the WHO and CSSE websites.

## Code availability

All codes used to generate the main results in this original manuscript are without restrictions to access and they were provided in Supplementary material.
