# Supplementary material for: Estimating the COVID-19 prevalence and mortality using a novel data-driven hybrid model based on ensemble empirical mode decomposition
Source: Sci Rep. 2021 Nov 1;11:21413. doi: 10.1038/s41598-021-00948-6 (PMC8560776; doi:10.1038/s41598-021-00948-6)
Supplement: Supplementary file 1 — Supplementary Information. [file 41598_2021_948_MOESM1_ESM.docx]

**Supplementary material:** **Estimating the** **COVID-19** **prevalence and mortality using a novel data-driven hybrid model based on ensemble empirical mode decomposition**

Yongbin Wang^1^^¶,*^, Chunjie Xu^2¶^, Sanqiao Yao^1^, Lei Wang^3^, Yingzheng Zhao^1^, Jingchao Ren^1^, Yuchun Li^1^

^1^ Department of Epidemiology and Health Statistics, School of Public Health, Xinxiang Medical University, Xinxiang, Henan Province, P.R. China

^2^ Department of Occupational and Environmental Health, School of Public Health, Capital Medical University, Beijing, P.R. China

^3^ Center for Musculoskeletal Surgery, Charité–Universitätsmedizin Berlin, Corporate Member of Freie Universität Berlin, Humboldt–Universität zu Berlin and Berlin Institute of Health, Berlin, Germany

^*^ Corresponding author: Yongbin Wang (wyb[who@163.com](mailto:wybwho@163.com)), No. 601 Jinsui road, Hongqi district, Xinxiang city, Henan province, 453003, P.R. China; Tel: +86-373-3831646

^¶^ These authors contributed equally to this paper

**The NARANN code used in our experiments**

% S – The target time series.

T = tonndata(S,true,false);

% Choose the 'trainlm' Training Function as it is usually fastest.

trainFcn = 'trainlm'; % Levenberg-Marquardt backpropagation.

% Create an NARANN model

feedbackDelays = 1:6;

hiddenLayerSize = 16;

net = narnet(feedbackDelays,hiddenLayerSize,'open',trainFcn);

% Choose Feedback Pre/Post-Processing Functions

% Settings for feedback input are automatically applied to feedback output

net.input.processFcns = {'removeconstantrows','mapminmax'};

% Prepare the Data for Training and Simulation

% The function PREPARETS prepares timeseries data for a particular network, shifting time by the minimum amount to fill input states and layer states..

[x,xi,ai,t] = preparets(net,{},{},T);

% Setup Division of Data for Training, Validation, Testing

net.divideFcn = 'divideblock'; % Divide data

net.divideMode = 'time'; % Divide up every sample

net.divideParam.trainRatio = 80/100;

net.divideParam.valRatio = 10/100;

net.divideParam.testRatio = 10/100;

% Choose 'mse’ as the Performance Function

net.performFcn = 'mse'; % Mean Squared Error

% Choose Plot Functions

net.plotFcns = {'plotperform','plottrainstate', 'ploterrhist', ... 'plotregression', 'plotresponse', 'ploterrcorr', 'plotinerrcorr'};

% Train the model

[net,tr] = train(net,x,t,xi,ai);

% Test the model

y = net(x,xi,ai);

e = gsubtract(t,y);

performance = perform(net,t,y)

% Recalculate Training, Validation and Test Performance

trainTargets = gmultiply(t,tr.trainMask);

valTargets = gmultiply(t,tr.valMask);

testTargets = gmultiply(t,tr.testMask);

trainPerformance = perform(net,trainTargets,y)

valPerformance = perform(net,valTargets,y)

testPerformance = perform(net,testTargets,y)

% View the Network

% view(net)

% Plots

%figure, plotperform(tr)

%figure, plottrainstate(tr)

%figure, ploterrhist(e)

figure, plotregression(t,y)

%figure, plotresponse(t,y)

figure, ploterrcorr(e)

%figure, plotinerrcorr(x,e)

% Closed Loop Network and prepared to make a multi-step ahead prediction

netc = closeloop(net);

netc.name = [net.name ' - Closed Loop'];

%view(netc)

[xc,xic,aic,tc] = preparets(netc,{},{},T);

yc = netc(xc,xic,aic);

closedLoopPerformance = perform(net,tc,yc)

% 12-data ahead forecast

[x1,xio,aio,t] = preparets(net,{},{},T);

[y1,xfo,afo] = net(x1,xio,aio);

[netc,xic,aic] = closeloop(net,xfo,afo);

[y2,xfc,afc] = netc(cell(0,12),xic,aic);

% Further predictions can be made by continuing simulation starting with the final input and layer delay states, xfc and afc.


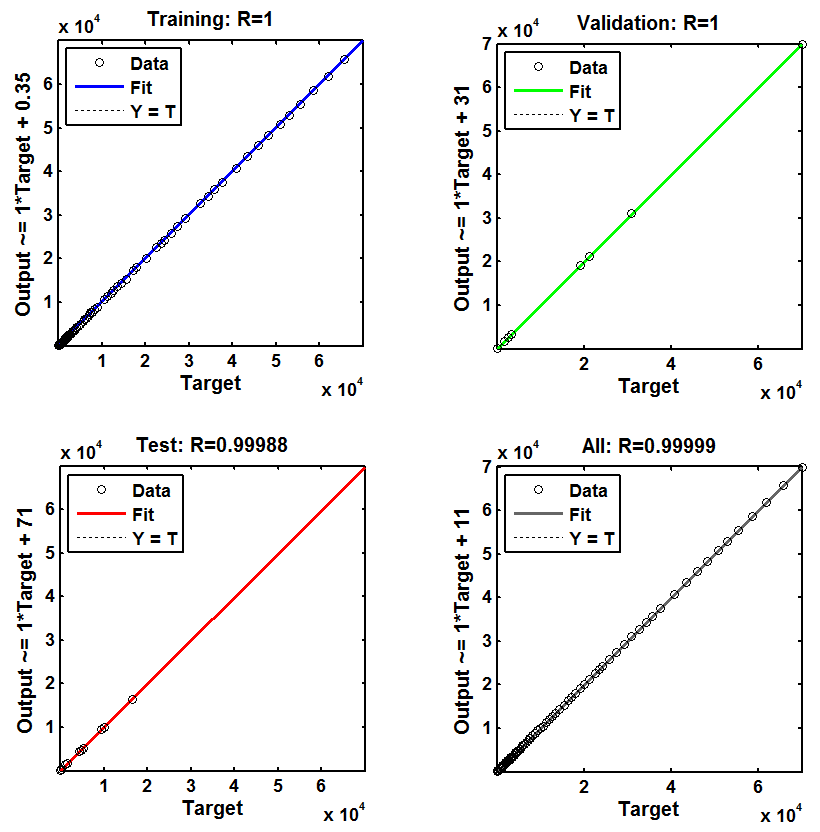


**Figure S1.** Regression plot showing the R values for the training, validation and test subsets of the prevalence data in South Africa. Regression R Values measure the correlation between outputs and targets. An R value of 1 means a close relationship, 0 indicates a random relationship. So the best NARANN produced a good approximation to the original prevalence data in South Africa.


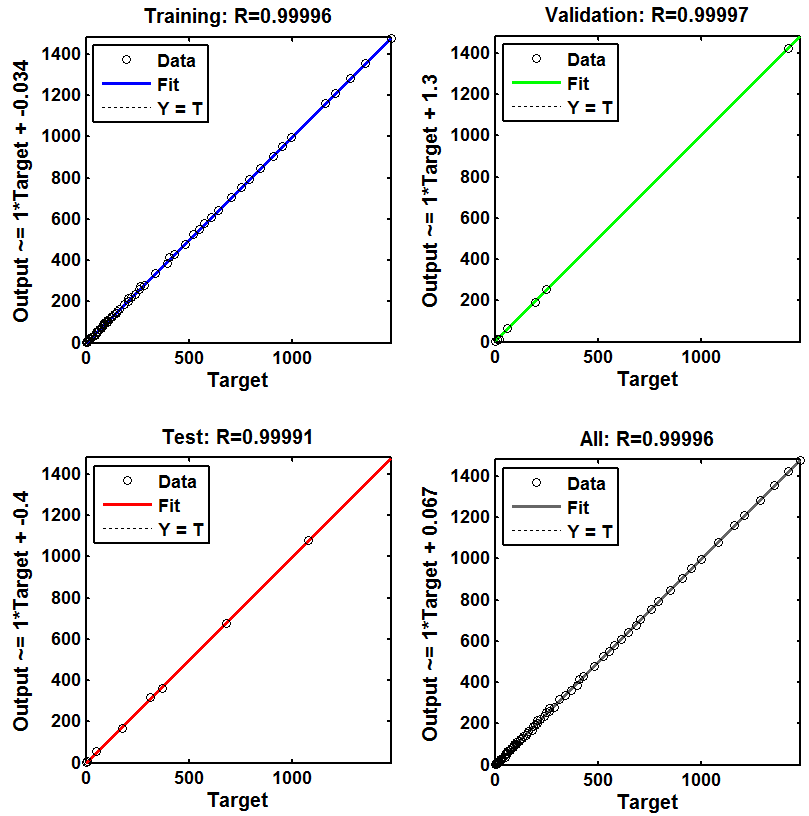


**Figure S2.** Regression plot showing the R values for the training, validation and test subsets of the mortality data in South Africa. Regression R Values measure the correlation between outputs and targets. An R value of 1 means a close relationship, 0 a random relationship. So the best NARANN produced a good approximation to the original mortality data in South Africa.


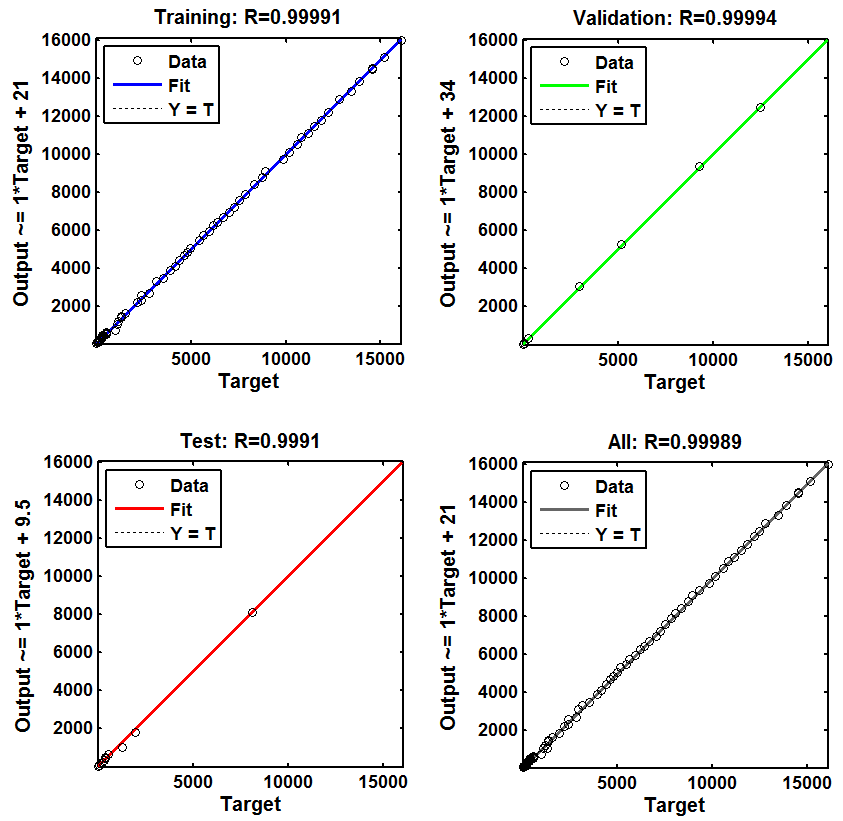


**Figure S3.** Regression plot showing the R values for the training, validation and test subsets of the prevalence data in Nigeria. Regression R Values measure the correlation between outputs and targets. An R value of 1 means a close relationship, 0 a random relationship. So the best NARANN produced a good approximation to the original prevalence data in Nigeria.


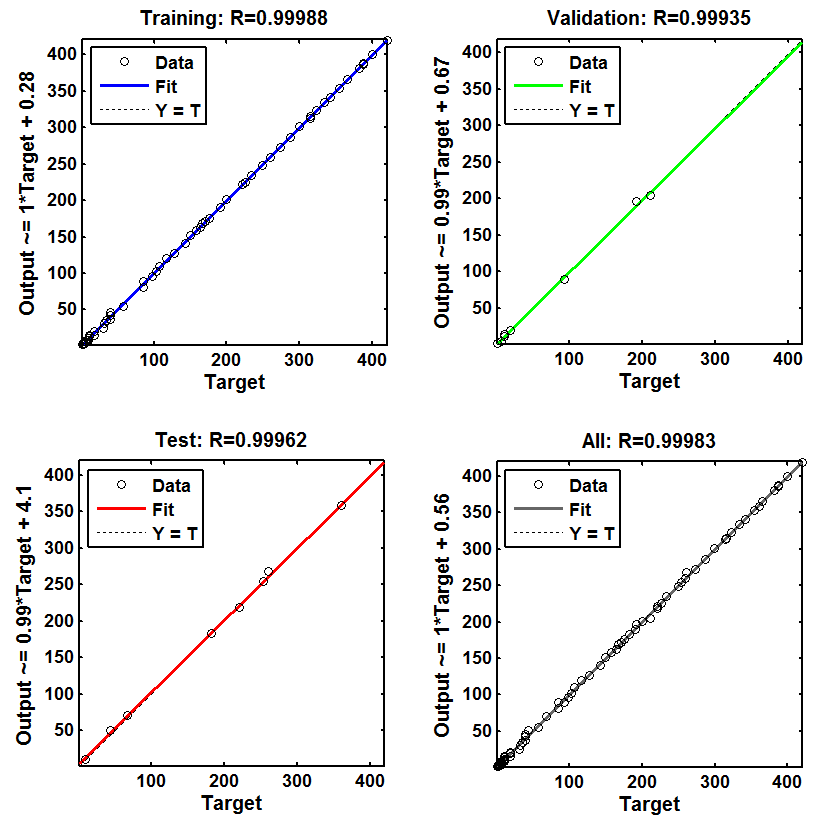


**Figure S4.** Regression plot showing the R values for the training, validation and test subsets of the mortality data in Nigeria. Regression R Values measure the correlation between outputs and targets. An R value of 1 means a close relationship, 0 a random relationship. So the best NARANN produced a good approximation to the original mortality data in Nigeria.


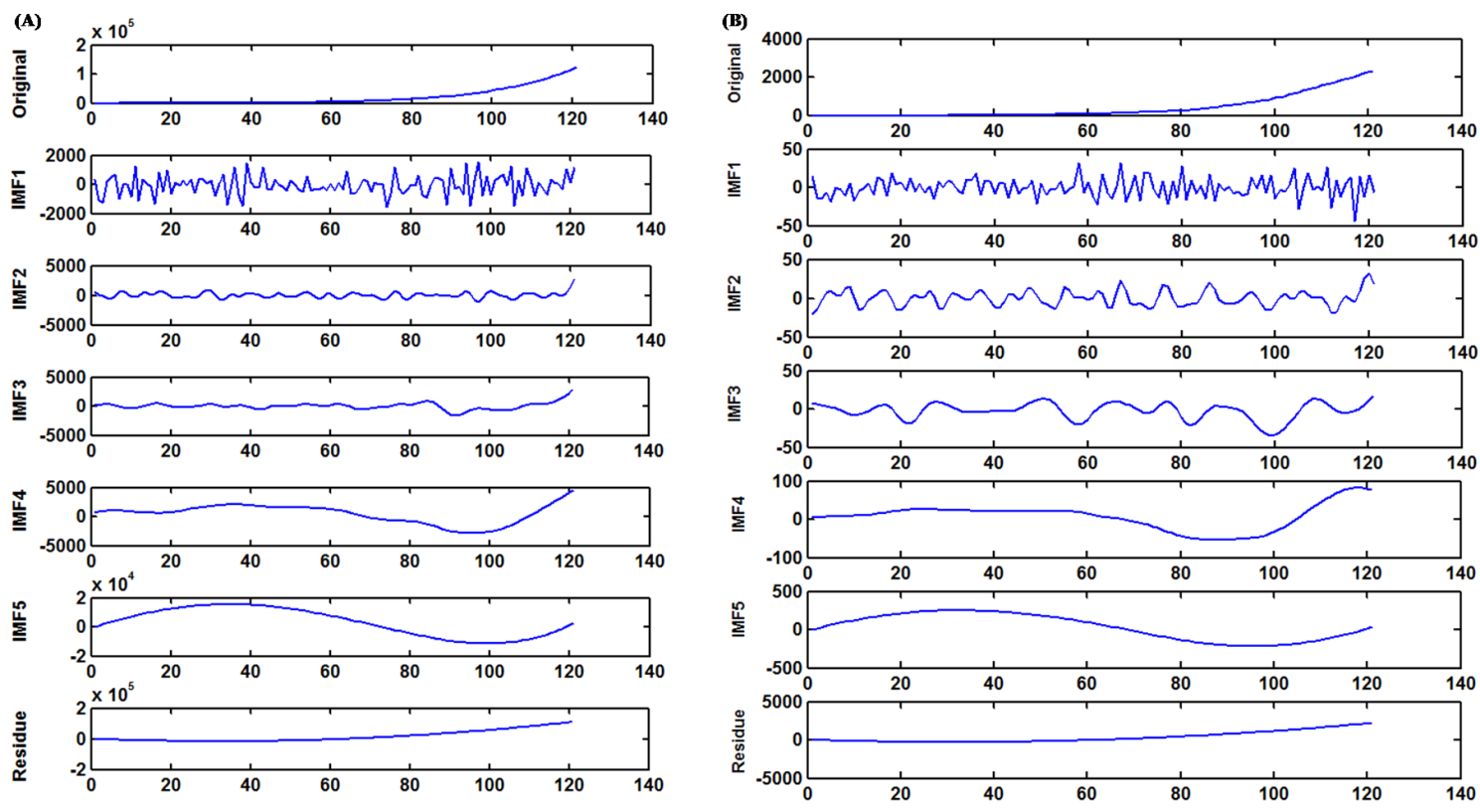


**Figure S5.** Intrinsic Mode Functions (IMFs) subseries via decomposing the original prevalence and mortality time series in South Africa. (A) The resulting IMFs subseries by decomposing the prevalence series in South Africa; (B) The resulting IMFs subseries by decomposing the mortality series in South Africa.


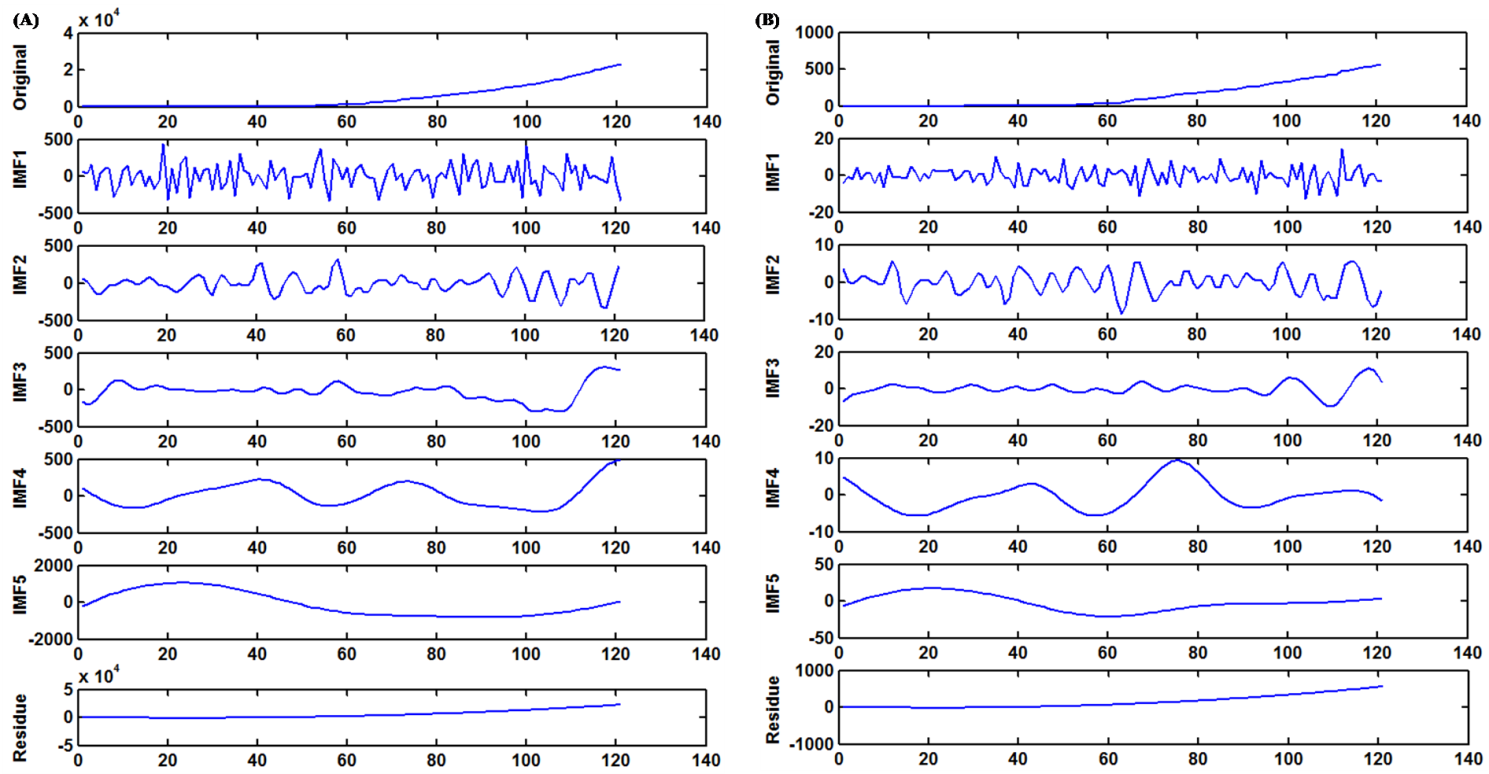


**Figure S6.** Intrinsic Mode Functions (IMFs) subseries via decomposing the original prevalence and mortality time series. (A) The resulting IMFs subseries by decomposing the prevalence series in Nigeria; (B) The resulting IMFs subseries by decomposing the mortality series in Nigeria.

| **Degree of difference** | **South Africa** | | **Nigeria** | |
| --- | --- | --- | --- | --- |
|  | **Statistics** | ***p*** | **Statistics** | ***p*** |
| **Prevalence data** | | | | |
| Actual series | 7.374 | 1.000 | 8.178 | 1.000 |
| First-order difference | 3.161 | 1.000 | -0.382 | 0.491 |
| Second-order difference | -7.665 | <0.001 | -10.957 | <0.001 |
| **Mortality data** | | | | |
| Actual series | 8.450 | 1.000 | 6.298 | 1.000 |
| First-order difference | -0.250 | 0.534 | -2.100 | 0.037 |
| Second-order difference | -12.645 | <0.001 | -10.281 | <0.001 |

**Table S1.** Augmented Dickey–Fuller (ADF) test for the original series and differenced series in South Africa and Nigeria.

| **Country** | **Model** | **Parameters** | **Estimates** | **Standard Error** | **t** | ***p*** | **R^2^** | **Stationary R^2^** | **Normalized BIC** | **Box-Ljung Q** | |
| --- | --- | --- | --- | --- | --- | --- | --- | --- | --- | --- | --- |
|  |  |  |  |  |  |  |  |  |  | **Statistics** | ***p*** |
| South Africa | Residue of the prevalence data | | | | | | | | | | |
|  | ARIMA(0,4,2) | MA1 | -1.019 | 0.084 | -12.164 | <0.001 | 1.000 | 0.493 | -13.179 | 5.616 | 0.992 |
|  |  | MA2 | -0.536 | 0.084 | -6.401 | <0.001 |  |  |  |  |  |
|  | Residue of the mortality data | | | | | | | | | | |
|  | ARIMA(1,3,0) | AR1 | 1.000 | 2.14E-05 | 46715.700 | <0.001 | 1.000 | — | -21.213 | 0.004 | 1.000 |
| Nigeria | Residue of the prevalence data | | | | | | | | | | |
|  | ARIMA(0,4,2) | MA1 | -0.803 | 0.097 | -8.293 | <0.001 | 1.000 | 0.382 | -16.364 | 3.248 | 1.000 |
|  |  | MA2 | -0.194 | 0.097 | -1.995 | 0.049 |  |  |  |  |  |
|  | Residue of the mortality data | | | | | | | | | | |
|  | ARIMA(1,4,0) | AR1 | 0.662 | 0.073 | 9.083 | <0.001 | 1.000 | 0.426 | -24.307 | 8.47 | 0.955 |

ARIMA Autoregressive integrated moving average method, BIC Bayesian information criterion, AR autoregressive method, MA moving average method.

**Table S2.** The identified parameters of the best-fitting ARIMA models for the decomposed residue of the COVID-19 the prevalence and mortality in South Africa and Nigeria.

| **Country** | **Model** | **Parameters** | **Estimates** | **Standard Error** | **t** | ***p*** | **R^2^** | **Stationary R^2^** | **Normalized BIC** | **Box-Ljung Q** | |
| --- | --- | --- | --- | --- | --- | --- | --- | --- | --- | --- | --- |
|  |  |  |  |  |  |  |  |  |  | **Statistics** | ***p*** |
| South Africa | Residue of the prevalence data | | | | | | | | | | |
|  | ARIMA(2,3,0) | AR1 | 0.682 | 0.121 | 5.614 | <0.001 | 1.000 | — | -11.840 | 0.002 | 1.000 |
|  |  | AR2 | 0.318 | 0.121 | 2.621 | 0.010 |  |  |  |  |  |
|  | Residue of the mortality data | | | | | | | | | | |
|  | ARIMA(0,4,2) | MA1 | -0.923 | 0.079 | -11.743 | <0.001 | 1.000 | 0.506 | -20.478 | 13.062 | 0.668 |
|  |  | MA2 | -0.547 | 0.079 | -6.958 | <0.001 |  |  |  |  |  |
| Nigeria | Residue of the prevalence data | | | | | | | | | | |
|  | ARIMA(0,4,1) | MA1 | -0.702 | 0.067 | -10.503 | <0.001 | 1.000 | 0.353 | -16.363 | 30.184 | 0.025 |
|  | Residue of the mortality data | | | | | | | | | | |
|  | ARIMA(1,4,1) | AR1 | 0.586 | 0.084 | 6.963 | <0.001 | 1.000 | 0.676 | -24.096 | 4.650 | 0.997 |
|  |  | MA1 | -0.640 | 0.081 | -7.894 | <0.001 |  |  |  |  |  |

ARIMA Autoregressive integrated moving average method.

**Table S3.** The identified parameters of the best-fitting ARIMA models for the decomposed residue of the COVID-19 the prevalence and mortality in South Africa and Nigeria.

| **Country** | **Target series** | **Hidden units** | **Delays** | **MSE** | | | **R** | | | |
| --- | --- | --- | --- | --- | --- | --- | --- | --- | --- | --- |
|  |  |  |  | **Training** | **Validation** | **Testing** | **Training** | **Validation** | **Testing** | **overall** |
| South Africa | **Prevalence data** | | | | | | | | | |
|  | IMF1 | 15 | 5 | 115589.604 | 411887.079 | 1400674.720 | 0.874 | 0.637 | 0.627 | 0.731 |
|  | IMF2 | 16 | 6 | 22577.000 | 21814.837 | 40738.516 | 0.954 | 0.955 | 0.929 | 0.950 |
|  | IMF3 | 15 | 5 | 253.368 | 1277.728 | 986.221 | 1.000 | 1.000 | 0.996 | 0.999 |
|  | IMF4 | 14 | 4 | 338.170 | 338.492 | 565.800 | 1.000 | 1.000 | 1.000 | 1.000 |
|  | IMF5 | 14 | 5 | 344.385 | 326.357 | 919.864 | 1.000 | 0.999 | 1.000 | 1.000 |
|  | **Mortality data** | | | | | | | | | |
|  | IMF1 | 17 | 5 | 9.331 | 261.687 | 192.832 | 0.969 | 0.648 | 0.392 | 0.842 |
|  | IMF2 | 16 | 5 | 7.098 | 17.195 | 11.398 | 0.967 | 0.913 | 0.967 | 0.958 |
|  | IMF3 | 15 | 5 | 9.185 | 10.924 | 11.017 | 0.952 | 0.943 | 0.930 | 0.950 |
|  | IMF4 | 15 | 4 | 7.800 | 5.895 | 17.086 | 0.957 | 0.955 | 0.953 | 0.955 |
|  | IMF5 | 15 | 4 | 6.806 | 9.096 | 13.746 | 0.965 | 0.925 | 0.943 | 0.959 |
| Nigeria | **Prevalence data** | | | | | | | | | |
|  | IMF1 | 17 | 5 | 6809.328 | 9263.409 | 51728.813 | 0.881 | 0.794 | 0.380 | 0.784 |
|  | IMF2 | 16 | 5 | 737.123 | 2566.846 | 2286.683 | 0.972 | 0.954 | 0.923 | 0.962 |
|  | IMF3 | 15 | 5 | 13.665 | 58.480 | 7.555 | 1.000 | 0.998 | 1.000 | 0.999 |
|  | IMF4 | 16 | 6 | 14.100 | 43.463 | 16.453 | 1.000 | 0.999 | 1.000 | 0.999 |
|  | IMF5 | 15 | 5 | 11.456 | 20.964 | 26.564 | 1.000 | 0.999 | 1.000 | 1.000 |
|  | **Mortality data** | | | | | | | | | |
|  | IMF1 | 18 | 5 | 8.853 | 11.706 | 41.250 | 1.000 | 1.000 | 0.990 | 1.000 |
|  | IMF2 | 16 | 5 | 6.184 | 22.780 | 54.562 | 1.000 | 0.999 | 0.999 | 1.000 |
|  | IMF3 | 15 | 5 | 9.003 | 8.063 | 67.731 | 1.000 | 1.000 | 0.978 | 1.000 |
|  | IMF4 | 14 | 6 | 8.788 | 15.527 | 36.362 | 1.000 | 1.000 | 1.000 | 1.000 |
|  | IMF5 | 15 | 5 | 8.381 | 26.251 | 45.324 | 1.000 | 0.999 | 0.999 | 1.000 |

MSE mean square error.

**Table S4.** The identified parameters of the best EEMD-ARIMA-NARANN hybrid models for different target series.

| **Time** | **South Africa** | | | | | | **Nigeria** | | | | | |
| --- | --- | --- | --- | --- | --- | --- | --- | --- | --- | --- | --- | --- |
|  | **Prevalence** | | | **Mortality** | | | **Prevalence** | | | **Mortality** | | |
|  | **Forecast** | **Lower limit** | **Upper limit** | **Forecast** | **Lower limit** | **Upper limit** | **Forecast** | **Lower limit** | **Upper limit** | **Forecast** | **Lower limit** | **Upper limit** |
| 28/06/20 | 128338 | 128089 | 128589 | 2411 | 2407 | 2416 | 24240 | 24188 | 24293 | 587 | 586 | 588 |
| 29/06/20 | 133200 | 132803 | 133592 | 2490 | 2479 | 2501 | 24253 | 24167 | 24342 | 575 | 573 | 577 |
| 30/07/20 | 134730 | 134220 | 135285 | 2603 | 2591 | 2618 | 24704 | 24590 | 24807 | 574 | 572 | 576 |
| 01/07/20 | 139896 | 139233 | 140445 | 2630 | 2610 | 2651 | 25243 | 25075 | 25388 | 595 | 592 | 598 |
| 02/07/20 | 143221 | 142316 | 144012 | 2695 | 2679 | 2718 | 25696 | 25569 | 25852 | 616 | 610 | 621 |
| 03/07/20 | 147968 | 146552 | 149450 | 2731 | 2716 | 2761 | 26483 | 26334 | 26632 | 653 | 644 | 659 |
| 04/07/20 | 154724 | 153800 | 155461 | 2782 | 2764 | 2802 | 27320 | 27114 | 27523 | 662 | 654 | 669 |
| 05/07/20 | 157122 | 155972 | 158735 | 2870 | 2829 | 2888 | 27590 | 27292 | 27808 | 668 | 663 | 677 |
| 06/07/20 | 156013 | 153882 | 157610 | 2933 | 2909 | 2975 | 27748 | 27412 | 28162 | 684 | 670 | 692 |
| 07/07/20 | 156781 | 155158 | 158532 | 3031 | 3004 | 3069 | 28443 | 28077 | 28796 | 682 | 669 | 692 |
| 08/07/20 | 163588 | 160993 | 165633 | 3117 | 3054 | 3142 | 28804 | 28602 | 29434 | 696 | 682 | 709 |
| 09/07/20 | 171169 | 169164 | 173068 | 3166 | 3134 | 3205 | 30095 | 29534 | 30258 | 719 | 704 | 741 |
| 10/07/20 | 174072 | 171589 | 176037 | 3251 | 3215 | 3291 | 30990 | 30393 | 31246 | 750 | 735 | 765 |
| 11/07/20 | 171688 | 169933 | 174046 | 3350 | 3296 | 3414 | 31382 | 30844 | 32026 | 782 | 760 | 788 |
| 12/07/20 | 176570 | 173607 | 178476 | 3454 | 3384 | 3487 | 32136 | 31568 | 32641 | 788 | 775 | 804 |

**Table S5.** The next 15 day estimates for the prevalence and mortality data in South Africa and Nigeria based on the best EEMD-ARIMA-NARANN hybrid model.
